# Enhanced adsorptive removal of indigo carmine dye by bismuth oxide doped MgO based adsorbents from aqueous solution: equilibrium, kinetic and computational studies

**DOI:** 10.1039/d2ra02636h

**Published:** 2022-08-31

**Authors:** Fatima A. Adam, M. G. Ghoniem, Moussa Diawara, Seyfeddine Rahali, Babiker Y. Abdulkhair, M. R. Elamin, Mohamed Ali Ben Aissa, Mahamadou Seydou

**Affiliations:** Department of Chemistry, College of Science, Imam Mohammad Ibn Saud Islamic University (IMSIU) Riyadh 11432 Saudi Arabia Famohamedali@imamu.edu.sa; Laboratoire de Centre de Calcul de Modélisation et de Simulation (CCMS), DER de Physique de La Faculté des Sciences et Techniques (FST), Université des Sciences des Techniques et des Technologies de Bamako (USTTB-Mali) Bamako Mali; Department of Chemistry, College of Science and Arts, Qassim University Ar Rass Saudi Arabia saif.rahali@gmail.com dalibenissa@gmail.com; Université de Paris, ITODYS, CNRS, UMR 7086 15 Rue J-A de Baïf 75013 Paris France

## Abstract

Novel doped MgO nanoadsorbents were effectively fabricated at various Bi_2_O_3_ doping concentrations (0, 2.5, 5 and 10%). DFT-D3 study showed that the doping is done by substitution of two magnesium atoms by two bismuth atoms with the creation of a vacancy of one Mg atom. TEM, SEM, EDX, BET, XRD, and FTIR were used to characterize the obtained nanostructures. The removal of indigo carmine (IC) dyes from wastewater by doped MgO nanoparticles is investigated. Experimental parameters such as the initial dye concentration, contact time, Bi_2_O_3_ doping concentration, and pH were optimized to enhance the adsorption capacity. Bi_2_O_3_ doped MgO prepared at 5% (MgOBi2) is the best adsorbent with a maximum IC adsorption capacity of 126 mg g^−1^ at a solution pH equal to 7.00 and contact time of 74 min. The results indicated that the adsorption process followed pseudo-second-order (PSO) reaction kinetics, and the Freundlich isotherm model gave a better goodness-of-fit than the linear Langmuir model. The FTIR study established that IC molecules are successfully adsorbed onto the surface of MgOBi2 *via* a chemisorption process.

## Abstract 2

The IC adsorption mechanism on the Bi_2_O_3_ doped MgO nanosorbents occurred through the chemisorption process.

## Graphical abstract

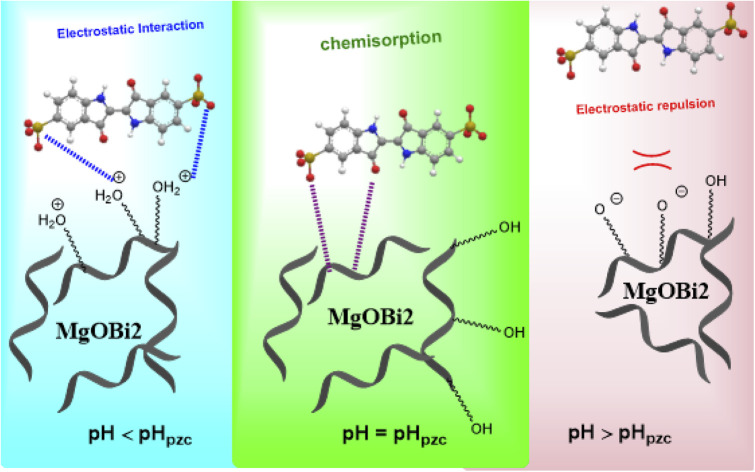

## 1. Introduction

Dyes and other organic pollutants have a tremendous effect on water resources and the total environment. Discharging of different organic pollutants such as pesticides, pharmaceuticals, oils, detergents, hydrocarbons, plastic, and dyes^1^ can negatively impact the quality of air, soil, and natural water resources through illegal discharge of industrial and domestic effluents. Nondegradable organic materials can disturb the food chain of the aquatic flora and fauna resulting in the death of fishes, algae, and other aquatic organisms leading to ecosystem collapse.^2^ The global annual production of natural and industrial dyes is about one million tons mainly consumed in textile, plastics, food, and other industries as coloring materials. Dyes can be toxic and carcinogenic in addition to their coloring properties.^3^ Color sustainability is one of the most desired characteristics of different industrial products such as textiles, leather, and plastics, hence the coloring materials, mainly dyes, should be stable compounds and nondegradable.^4^ This is a contradicted objective between the industrial sector and the environmental concerns bearing in mind that even a concentration of 1.0 ppm of dyes in the water can heavily impact its characteristics.^5^

Indigo carmine, acid blue, is an organic compound with a chemical name, 5,5′-indigodisulfonic acid sodium salt (C_16_H_8_N_2_Na_2_O_8_S_2_, 466.36 g mol^−1^),^5^ with two sulphonated groups and four aromatic rings.^6^ It is utilized as dyeing material for many purposes including textile coloring, cosmetics, printing, biological staining, dermatological and antibacterial agent, and as an additive to poultry feed.^7^ It is considered as a potent toxic to mammalian cells, irritant, recalcitrant in addition to a high coloring capacity for aqueous solutions.^8–10^

Protection of the environment from the devastating impacts of dyes and other pollutants is a great challenge facing humanity recently. Many water treatment methodologies are adopted worldwide to enhance the quality of natural water resources and to treat the liquid effluent emerging from different sectors. Photodegradation, electrocoagulation, electrochemical, flocculation–coagulation, ultra-filtration, and adsorption are among the most used techniques.^11–15^ Based on efficiency, scalability, low operation cost, simplicity, adsorption can be considered as the most preferred method for water treatment among all other techniques. The nature and amount of pollutants are the most essential factors for selecting the treatment method.^4^ Activated carbon is a widely used adsorbent in addition to different types of clays. Recently, many special sorbents were practiced including metal oxides and their composites. Availability and relative safety of magnesium and bismuth metals in addition to other properties, nominate them as potential candidates for wastewater treatment through adsorption and photocatalytic actions.^16,17^ Metal oxides nanomaterials with layered double hydroxides and hierarchical nanostructures exhibit excellent adsorption capabilities for heavy metals removal due to their high surface area. Magnesium oxide and its nanoparticles can be prepared through different routes, including chemical, physical and biological methods.^18^ Many researchers investigated magnesium oxide nanoparticles and their composites to explore their capacities in removing various water pollutants. Feng *et al.* prepared aluminum and magnesium oxide nanoparticle composite through a one-step microwave assisted-solvothermal method to remove arsenic and lead ions from water successfully.^19^ Biosynthesized magnesium oxide was used to treat chromium successfully and reduce other pollutants in a tannery effluent.^20^ Jamil *et al.* synthesized magnesium oxide nanoparticles with a size of 19 nm for Reactive Orange 122 and removal of Reactive Black 5 from water, they attained maximum adsorption capacities, *q*_max_, of 333.34 and 500 mg g^−1^ respectively.^21^ Ramesh *et al.* studied the interaction nature of the adsorption of indigo carmine on magnesium oxide, they found that the interaction followed Langmuir and Harkin–Jura isotherms at pH 7, confirming the occurrence of mono and multilayer adsorption simultaneously.^5^ Naga *et al.* used waste red mud from the alumina industry to remove indigo carmine from aqueous solutions, the maximum adsorption capacity was found to be 62 mg g^−1^, the adsorption mechanism followed the Langmuir model with mono-layer adsorption and endothermic and spontaneous.^8^ Many magnesium oxides nanomaterials were fabricated to enhance and add new properties to the adsorbent. Induni *et al.* prepared a composite made of magnesium oxide and granular activated carbon to adsorb hydrogen sulphide gas, H_2_S, the adsorption efficiency of the magnesium composite increased five times that of the granulated activated carbon alone.^22^ Magnesium bismuth oxides composites with silver iodide were prepared by Dai *et al.*, as a photocatalytic active agent to remove some dyes from water, the composite showed a performance of 92.3–99.5% degradation within 10 min under visible light irradiation.^23^ The use of density functional theory has become increasingly common among chemists, providing them with a useful tool to study the mechanism of interaction between two chemical species^24,25^ in particular the adsorption of molecules on the surface of a solid,^26–29^ thus the rationalization of the doping mechanism of some metal oxides.^30–32^

The current study aims to fabricate magnesium bismuth oxide nanoparticles in different proportions and explore their removal capacities as environmentally safe sorbent for removing indigo carmine hazardous dye. The removal parameters will be optimized, and the kinetics and thermodynamic behavior will be investigated. To rationalize the MgO doping mechanism by substitution between Mg^2+^ and Bi^3+^, *ab initio* calculations based on the Density Functional Theory (DFT) as implemented in the Vienna *Ab initio* Simulation Package (VASP) were used.

## 2. Materials and methods

### 2.1. Chemicals

Bismuth(iii) nitrate pentahydrate, magnesium nitrate hexahydrate, nitric acid (65%), ethanol (absolute), sodium hydroxide, sodium chloride, hydrochloric acid, and ammonium hydroxide were supplied commercially from Merck company and utilized without additional purification process. The IC concentrations (10 to 100 ppm) were obtained by diluting the IC stock solution (200 ppm).

### 2.2. Nanoparticles synthesis

The co-precipitation method was utilized to synthesize Bi_2_O_3_ doping MgO nanoparticles. To fabricate 2.5% by weight of Bi_2_O_3_–MgO nanoparticles, 0.534 g of Bi(NO_3_)_2_·5H_2_O and 64.0 g of Mg(NO_3_)_2_·6H_2_O were added to 20 mL of nitric acid solution (1.12 M). After stirring for 15 min, NH_4_OH solution (33%) was added to the mixture until pH value 11 was reached. The solution was stirred for six hours at room temperature. Then, the mixture was filtered and washed with distilled water and absolute ethanol several times. The obtained powder was dried in an oven at 80 °C for 2.0 h and calcined at 450 °C for two hours.^33^ The other percentages by weight (0, 5, and 10%) were prepared similarly. The as-fabricated Bi_2_O_3_ doped MgO nanoparticles with different proportions of Bi_2_O_3_ (0, 2.5, 5, and 10%) were symbolized as MgOBi0, MgOBi1, MgOBi2, and MgOBi3, respectively.

### 2.3. Characterization of the nano-adsorbents

The morphology of the as-fabricated nanopowders was analyzed by field emission scanning electron microscopy (JEOL JEM-6700F apparatus) and transmission electron microscope (Tecnai G20-USA). X-ray diffraction (XRD) was used to determine the structural properties using a Rigaku Mini Flex 600 (Tokyo, Japan) diffractometer equipped with a CuK radiation source (*λ* = 1.5417). The Brunauer, Emmett and Teller (BET) formula, as well as Lippens and de Boer's *t*-plot approach, were used to determine the surface area and porosity of nanopowders. The vibration modes of pure and Bi_2_O_3_ doped MgO nanoparticles before and after IC dye adsorption were determined using a JASCO FT-IR spectrometer.

### 2.4. Adsorption study

Batch removal studies were carried out in 50 mL screw-top bottles containing 25 mL IC dye solutions to elucidate the influence of the operational parameters, including the pH solution (2–11), initial IC dye concentration (10–100 ppm), and contact time (2–210 min). The mixture was stirred at 400 rpm for 3.0 h. Following each experiment, the mixture was centrifuged, and the residual concentrations of IC dye were determined using a LABOMED: UVS-2800 spectrophotometer at a maximum wavelength of 610 nm.

The equilibrium quantity of IC dye adsorbed per unit mass of Bi_2_O_3_ doped MgO nanoparticles was measured applying eqn (1):^34^1
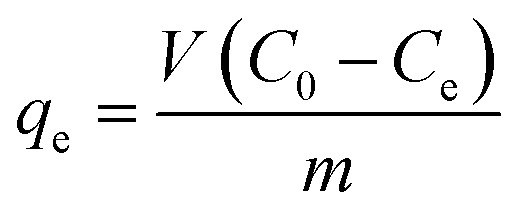


The kinetic experiment's volume and initial concentration of dyes were 250 mL and 20 ppm, respectively. The Bi_2_O_3_ doped MgO nanoparticles mass was 50 mg. 5 mL of the suspension was removed and centrifuged at specific time intervals to determine the residual IC dye concentration. A related equation was utilized to calculate at time *t* the quantity sorbed *q*_*t*_ (mg g^−1^):2
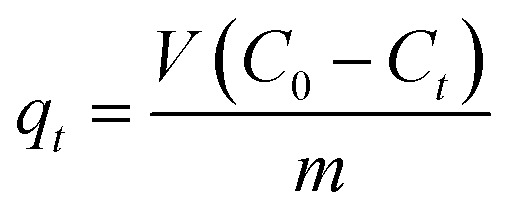


### 2.5. Computational methodology

The Vienna *Ab initio* Simulations Package^35^ was used to optimize the structures of MgO, and Bi_2_O_3_ doped MgO isolated and adsorbed to determine their adsorption energies. Periodic structures were built using Model View software. The slab model (200) was used with a vacuum size of 30 Å along the *z*-axis. The generalized gradient approximation (GGA) method with the Perdew–Burke–Ernzerhof of (PBE) functional^36^ was used to solve the Kohn–Sham equations. The projector augmented-wave method (PAW) was used to describe electron–ion interactions. The convergence of the plane-wave expansion was obtained with a cutoff of 500 eV. Sampling in the Brillouin zone was performed on a grid of (331) *k*-points for the geometry optimizations. The dispersion contribution was added using the DFT-D3 approach proposed by Grimme *et al.*^37^

## 3. Results and discussion

### 3.1. Nanomaterials characterizations

**Fig. 1:**
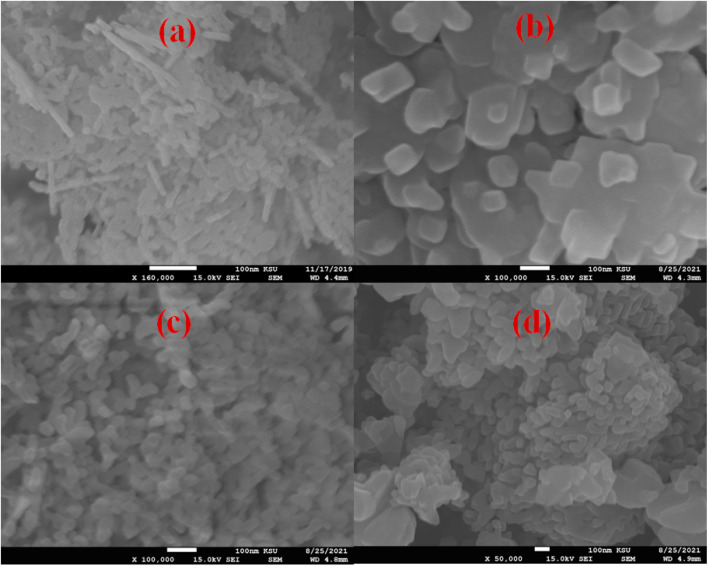
SEM images of (a) MgOBi0, (b) MgOBi1, (c) MgOBi2, and (d) MgOBi3.

To highlight the morphological and structural properties of the synthesized nanomaterials, SEM and TEM micrographs of the produced nanomaterials are realized. Fig. 1a–d shows the micrographs obtained from the SEM investigation of pure MgO (MgOBi0) and Bi_2_0_3_ doped MgO (MgOBi1, MgOBi2, and MgOBi3). As illustrated in Fig. 1a, the rod-like structure was found in the SEM image of MgOBi0. The sheet structure was observed in the SEM images of doped MgO, as displayed in Fig. 1b–d.

**Fig. 2:**
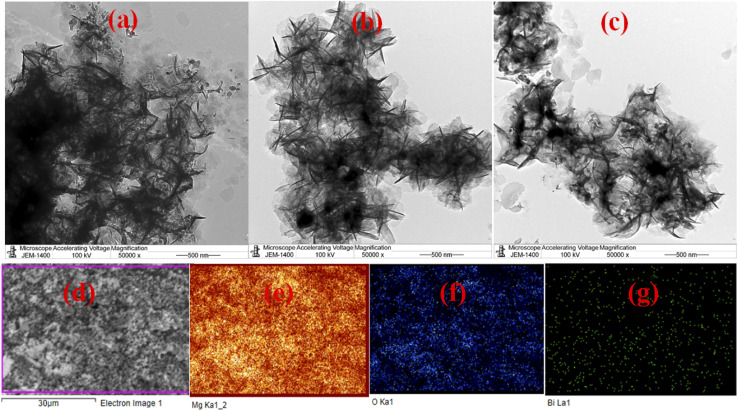
TEM images of (a) MgOBi1, (b) MgOBi2, and (c) MgOBi3, and. X-ray elemental mapping of (e) Mg−Ka, (f) O−Ka, and (g) Bi–La of the SEM MgOBi3 selected area (d).

Additionally, the TEM was used to study the shape of Bi_2_O_3_ doped MgO. TEM micrographs of doped MgO were shown in Fig. 2a–c. The small-sized nanoparticles were observed with a length of about 100 nm. The TEM images for the three nanocomposites may be lookalike, which can be attributed to the slight similarity in their detailed morphology due to the domination of MgO. Elemental mapping analysis of MgOBi3 shown in Fig. 2e–g reveals that Bi_2_O_3_ doped MgO possesses all elements. *i.e.*, Mg, Bi, and O elements.

**Fig. 3:**
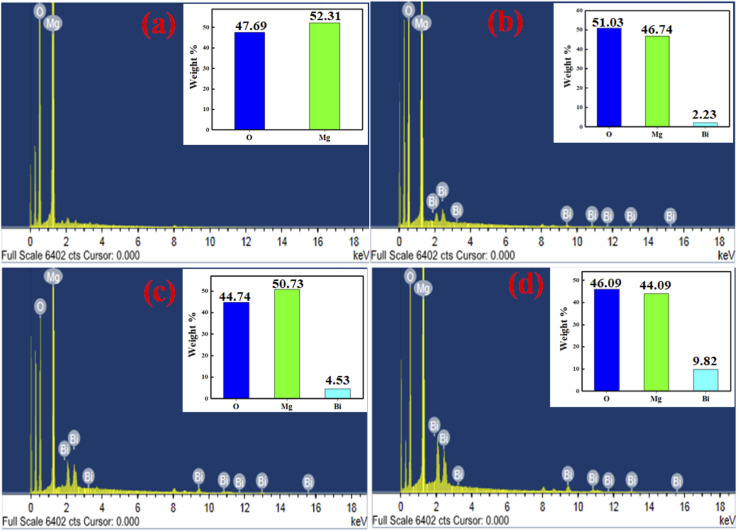
EDX images of (a) MgOBi0, (b) MgOBi1, (c) MgOBi2, and (d) MgOBi3.

The EDX spectrums of pure MgO and Bi_2_O_3_ doped MgO nanoparticles are shown in Fig. 3a–d, where the spectrums confirm the presence of Mg, O, and Bi peaks in the absence of any other ingredient. Additionally, Fig. 3a and b shows the surface percentage compositions of Bi_2_O_3_ loaded on MgO. The weight percentages of all the elements (Mg, Bi, and O) in MgOBi0 (Fig. 3a), MgOBi1 (Fig. 3b), MgOBi2 (Fig. 3c), and MgOBi3 (Fig. 3d) are verified by EDS spectral peaks of these samples.

**Fig. 4:**
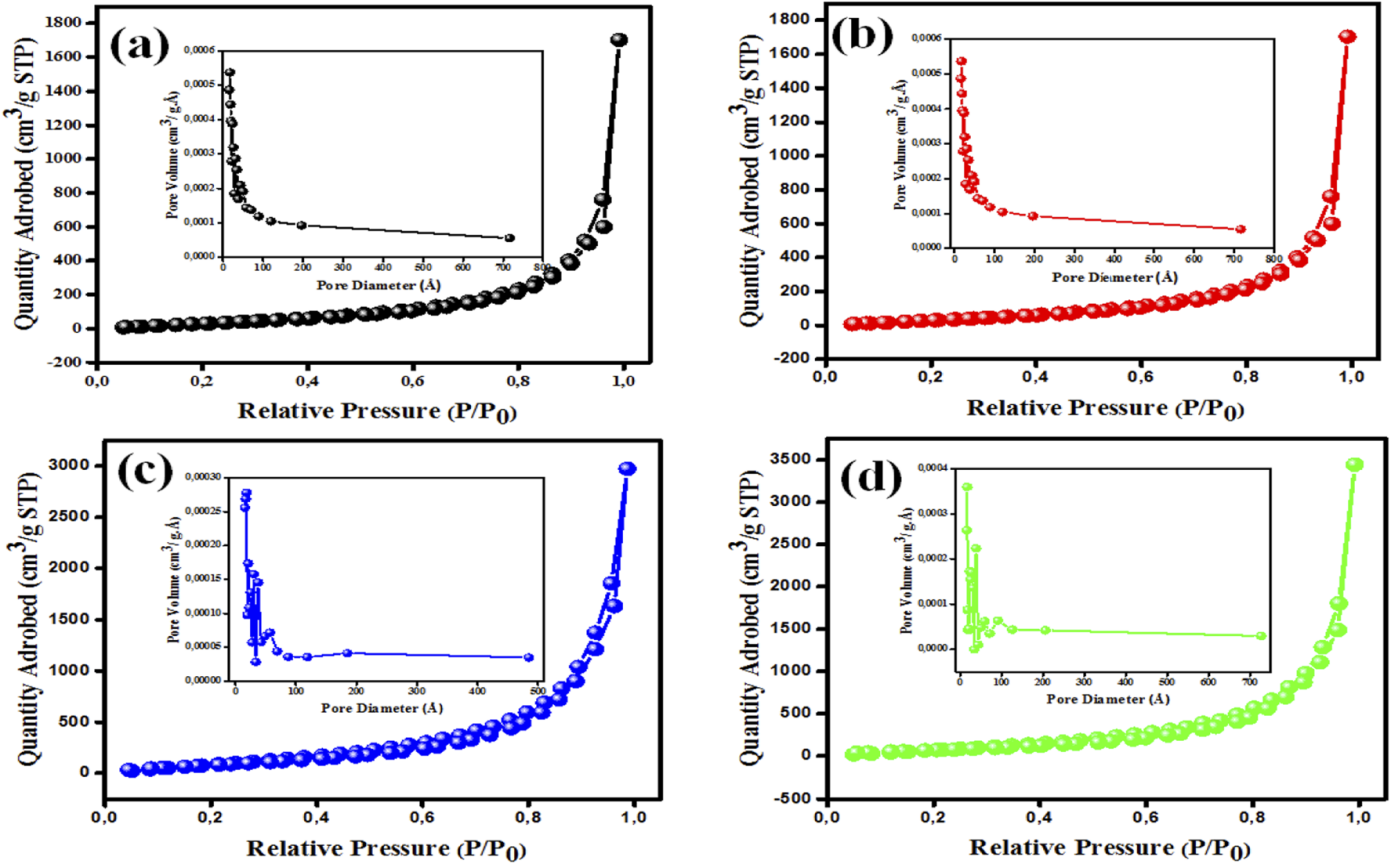
BET isotherms with pore size distribution (inset) of (a) MgOBi0, (b) MgOBi1, (c) MgOBi2, and (d) MgOBi3.

The BET isotherms for the developed nanomaterials with their pore size distributions are shown in Fig. 4. The N_2_ sorption isotherms are classified as Langmuir type IV by the IUPAC,^38^ which denotes the presence of mesoporous nanostructures.^38,39^ The BET isotherms of MgOBi0 and MgOBi1 showed some similarities attributed to minor doping amount and the domination of MgO properties; conversely, the increase of Bi_2_O_3_ in MgOBi2 and MgOBi3 showed noticeable alteration. The variation of pore size distribution illustrated in Fig. 4c and d indicated a different conformation take place as the Bi_2_O_3_ increased which is inline with the SEM findings. As demonstrated in Table 1, the pure MgO nanoparticles have a BET surface area of only 5.307 m^2^ g^−1^. Adding Bi_2_O_3_ to MgO changed the *S*_BET_ to 8.61, 12.215, and 5.160 m^2^ g^−1^, respectively, for MgOBi1, MgOBi2, and MgOBi3. Additionally, the pore volume and diameter varied between 0.029 and 0.082 cm^3^ g^−1^ and 1.7 and 1.8 nm, respectively. The obtained surface area and pore volume values nominated the MgOBi2 to possess the best adsorption among the other nanocomposites. Based on the obtained data, it can be concluded that the addition of Bi_2_O_3_ to MgO affects the pore size distribution and surface area.

**Table 1:** Pore volume and BET surface area properties of prepared nanomaterials

Nanomaterials	Pore volume (cm^3^ g^−1^)	BET surface area (m^2^ g^−1^)	Pore radius (Å)
MgOBi0	0.029	5.307	18.411
MgOBi1	0.041	8.691	17.12
MgOBi2	0.082	12.215	17.108
MgOBi3	0.029	5.160	18.401

**Fig. 5:**
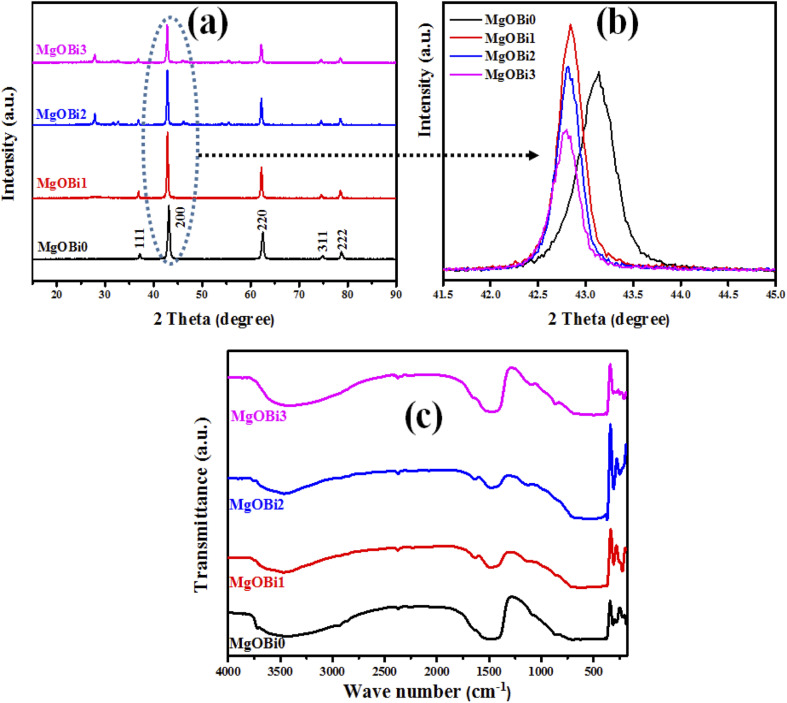
(a) XRD pattern, (b) principal XRD peak alterations, and (c) FTIR spectra for MgOBi0, MgOBi1, MgOBi2, and MgOBi3.

To examine the effect of Bi_2_O_3_ doping on the structural properties of the MgO phase, XRD of pure and doped MgO samples were performed. The results of MgO loaded with various Bi_2_O_3_ ratios are given in Fig. 5. Spotted peaks at 2*θ*° = 78.8°, 74.7°, 62.5°, 43.10°, and 37° were in accord with (222), (311), (220), (200), and (111) and planes demonstrating that MgO had cubic structure agreeing with (JCPDS 75-1525).^40^ Upon doping, the diffraction peaks were locomoted toward lower angles. For example, the principal peak (200) shifts by 0.30, 0.34, and 0.36° for MgOBi1, MgOBi2, and MgOBi3, respectively (Fig. 5b). This change for doped MgO can be related to the substitution of Mg ions (ionic radius = 0.74 Å) by Bi ions (ionic radius = 1.61 Å). The observed shift could result from the structural stresses induced by the increased ionic radius and the alteration of the MgO lattice properties.^41^ To interpret the experimental results and rationalize the Bi_2_O_3_ doped MgO mechanism, a theoretical study based on DFT calculations will be developed in the next section.

FTIR analysis has been undertaken to identify the chemical bonding and the purity of the manufactured compounds, see Fig. 5c. FTIR spectra showed broadband at 3465 cm^−1^ assigned to the OH stretching vibration,^42^ while the band at 1639 cm^−1^ is ascribed to the adsorbed water molecule's OH stretching mode.^43^ In addition, the characteristic band observed at 430 cm^−1^ is related to Mg–O vibration mode,^44^ demonstrating the formation of the MgO phase. Furthermore, the absorption bands in the FTIR spectra of doped MgO at 248 and 1108 cm^−1^ are associated with the vibrations of Bi–O bonds and the bending vibration of Bi=O, respectively.^45,46^

The average crystallite sizes of pure and Bi_2_O_3_ doped MgO nanoparticles were obtained from the full width at half maximum (FWHM) of the higher intensity peaks using Scherrer's formula (eqn (3)):^47^3
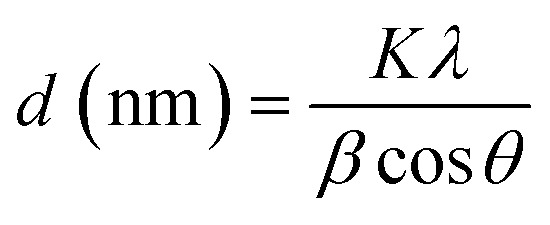
where *β* is the full width at half maximum (FWHM) of the diffraction peak (radians), *θ* is the Bragg diffraction angle, *λ* is the wavelength of X-ray radiation (1.5417 Å), and *K* is the shape factor (generally taken as 0.9). The crystal sizes of undoped and doped MgO nanocomposite samples with different Bi_2_O_3_ contents are given in Table 2.

**Table 2:** Average crystallite sizes of MgO and Bi_2_O_3_ doped MgO nanoparticles

Nanomaterials	FWHM (rad)			Crystallite size (nm)
	[200]	[220]	[222]	
MgOBi0	0.00847	0.00924	0.01019	193.11
MgOBi1	0.0056	0.00666	0.00784	292.07
MgOBi2	0.00533	0.0064	0.00738	306.84
MgOBi3	0.00585	0.00678	0.00801	279.16

### 3.2. IC dye adsorption investigation

#### 3.2.1 Effect of initial IC dye concentration and Bi_2_O_3_ doped concentration

**Fig. 6:**
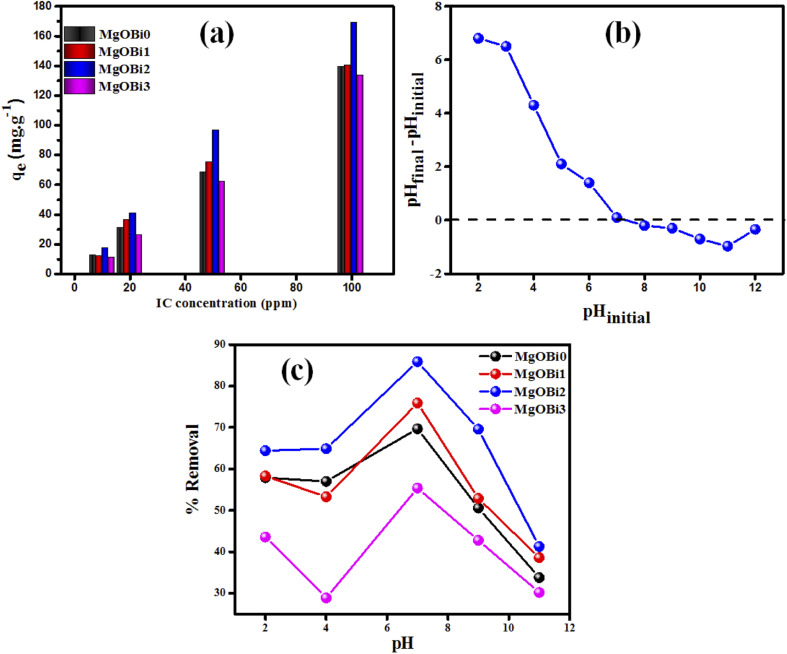
(a) Effect of initial IC concentration on the sorption by Bi_2_O_3_ doped MgO, (b) plot for the determination of pH_pzc_ for MgOBi2, (c) influence of pH on removal% of IC.

The initial dyes concentration is one of the considerable crucial factors influencing their adsorption onto the adsorbent. The impact of initial organic pollutant concentration was carried out in the range of 10–100 ppm, as shown in Fig. 6a. It was observed that the adsorption capacity increases significantly for all used nanomaterials when the IC dye concentration is raised. For example, the IC adsorption capacity increases significantly from 18.9 up to 169.2 mg g^−1^ for MgOBi2. It can be shown here that increasing the initial IC concentration generates an efficient force that overcomes any resistance to dye molecules' migration.

The surface area available for adsorption significantly affects the adsorption dyes process. To define the most effective nanomaterial for IC adsorption, doped MgO with varying Bi_2_O_3_ concentrations were examined as adsorbents for IC removal from solution. As shown in Fig. 6a, the amount of IC adsorbed increases as the Bi_2_O_3_ doping rate increases up to 5 percent and subsequently falls. The amount of Bi_2_O_3_ doped achieves saturation when the Bi_2_O_3_ level is 5 percent. MgOBi2 was found to have a maximum IC adsorption capacity due to its wide surface area, as shown in Table 1.

#### 3.2.2 Effect of pH

The pH value is a critical factor impacting the adsorbent's surface characteristics and the availability of appropriate functional groups on the dye molecules.^48^ The adsorbate molecules and adsorbents often contain a variety of surface functional groups that can be protonated/deprotonated in response to the medium pH, so modulating the electrostatic interaction between dyes and adsorbents and thereby increasing the effectiveness of pollutant elimination. The solutions were adjusted by adding HCl and/or NaOH dropwise while monitoring the pH value *via* a pH meter. In order to avoid any possibility of HCl evaporation, 0.01 g sorbent and 25 mL of IC dye solution (50 ppm) were stirred at the lowest tested temperature (25 °C). The point of zero charge (pH_pzc_) was employed to determine the zero charge of MgOBi2. Fig. 6b demonstrates that the pH_pzc_ of MgOBi2 was 7.01. The MgOBi2 nanomaterials' surface charge was zero at pH 7.01. The doped MgO will have a positively charged surface at a pH less than 7.01, and it will have a negative charge at a pH greater than pH_pzc_. The p*K*_a_ of IC is 12.6, and the IC structure is negatively charged at lower pH.^49^ Parallel experiments are conducted to determine the impact of pH on the IC dye removal efficiency. Fig. 6c illustrates the influence of altering the pH of IC dye solutions on the removal efficiency in the range of 2 to 11. It has been found that when pH rises, the percentage of IC removal boosts and reaches the maximum at pH equal to seven for all used nanomaterials. It is noted that MgOBi2 nanomaterials have the maximum percentage removal compared to other used nanomaterials. For pH values greater than 7, the rate of IC removal decreases due to the electrostatic repulsion between the negatively charged surface of nanoparticles and the negatively charged IC molecules. This result agrees with the work of Yazdi *et al.*^50^ They found that the electrostatic attraction responsible for the IC dye's adsorption onto magnetite nanoparticles will transform at high pH values into electrostatic repulsion between the anion compartment of the IC molecules and negatively charged function groups of nanoparticles. Additionally, Chaari *et al.* established that the electrostatic repulsion inhibits the IC dyes' adsorption at pH greater than pH_pzc_.^51^ Hence, the optimal pH of 7 was kept constant for future study.

### 3.3. Contact time and kinetic studies

The effect of equilibrium time for the IC adsorption onto utilized nanoparticles (MgOBi0, MgOBi1, MgOBi2, and MgOBi3) has been studied for a fixed IC starting concentration of 20 ppm during an agitation period of 2 and 210 min, see Fig. 10a. The IC removal is accelerated with the contact time and reaches the equilibrium within 60, 62, 74, and 66 min for MgOBi0, MgOBi1, MgOBi2, and MgOBi3, respectively. The adsorption rates increase significantly during the first few min of the process for all employed nanoparticles due to the accessibility of many active sites available on nanoparticles' surfaces. Finally, the concentration of the active sites declines upon reaching the equilibrium resulting in a lower sorption rate. Consequently, the IC molecules' removal remains almost unchanged.

**Fig. 7:**
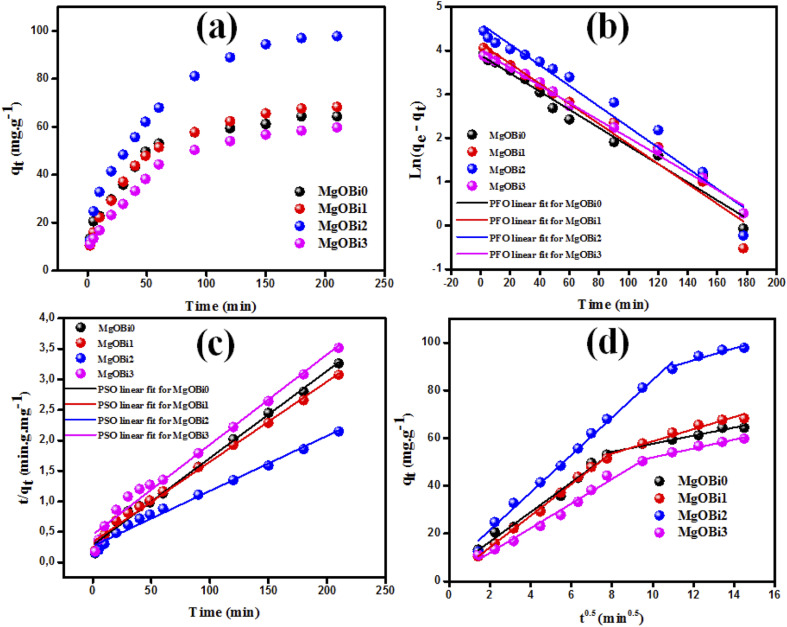
(a) Contact time, (b) PFO, and (c) PSO for IC adsorption onto employed nanoparticles and (d) intra-particle diffusion plots for IC sorption.

A kinetic study for the IC removal by fabricated nanoparticles has been carried out for a sorbent dose of 200 mg L^−1^ and [IC]_0_ = 20 ppm, while varying the contact time from 2 to 210 min. The relevant equations and the obtained results (kinetics parameters and the correlation coefficient *R*^2^) are given in Table 3. The linear pseudo-first-order (PFO), linear pseudo-second-order (PSO) and intra-particle diffusion models were used to assess the experimental data for IC dye adsorption onto doped and undoped MgO. PFO model is depicted in Fig. 7b. The slope of the plot between ln(*q*_e_ − *q*_*t*_) and adsorption time was used to calculate the kinetic parameters. The estimated *q*_e_ values significantly diverge from the experimental *q*_e_ values (Table 3) because the linear correlation coefficient *R*^2^ values for these plots are low. Fig. 7c illustrates a linear fitting of adsorption data using a PSO kinetic model. The excellent linear fit of (*t*/*q*_*t*_) *vs.* adsorption time with an *R*^2^ of 0.982–0.994 (Table 3) for various doped Bi_2_O_3_ concentrations indicated that the adsorption of IC dye onto the produced nanomaterials followed a PSO model. The good agreement between the observed and estimated *q*_e_ values further demonstrated that the PSO adsorption model fits the adsorption kinetics well.

**Table 3:** Kinetics models for IC adsorption by Bi_2_O_3_ doped MgO

Kinetics model	Kinetic equation	Parameter	MgOBi0	MgOBi1	MgOBi2	MgOBi3
PFO^57^	ln(*q*_e_ − *q*_*t*_) = ln *q*_e_ − *k*_1_*t*	*q*_e_ (mg g^−1^)	49.02	63.79	100.43	44.05
		*K*_1_ (min^−1^)	0.20	0.22	0.23	0.19
		*R* ^2^	0.97	0.96	0.96	0.98
PSO^57^	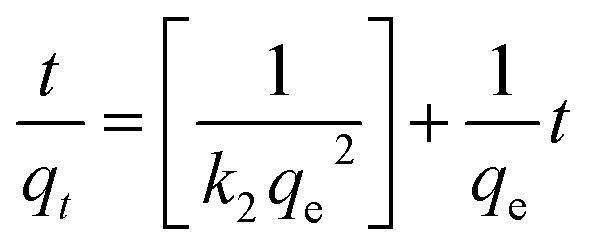	*q*_m_ (exp) (mg g^−1^)	64.50	68.80	101.71	59.80
		*q*_m_ (cal) (mg g^−1^)	70.07	76.04	110.74	67.70
		*K*_2_ (g mg^−1^ min^−1^) × 10^4^	7.17	5.17	3.04	4.81
		*h*_0_ (mg g^−1^ min^−1^)	3.52	2.98	3.72	2.19
		*t*_1/2_ (min)	19.72	25.43	29.70	30.70
		*R* ^2^	0.99	0.99	0.99	0.99
IPD^58^	*q*_*t*_ = *k*_dif_*t*^1/2^ + *C*	*K*_dif1_ (mg g^−1^ min^1/2^)	6.22	6.60	7.86	5.15
		*C*_1_	4.15	1.04	5.78	1.64
		*R* ^2^	0.98	0.99	0.99	0.98
		*K*_dif2_ (mg g^−1^ min^1/2^)	1.69	2.55	2.50	1.87
		*C*_2_	40.70	33.12	62.57	33.17
		*R* ^2^	0.96	0.96	0.92	0.97

The dyes molecules may be transferred from the solution bulk to the nanoparticles solid phase by the intra-particle diffusion/transport mechanism.^52,53^ Intra-particular diffusion is a restricting step in the adsorption mechanism. The possibility of intra-particular diffusion is established using the Weber and Morris diffusion pattern.^52,53^ The higher regression coefficients (Table 3) exhibit the efficacy of the intra-particle mode of diffusion for all tested nanomaterials. As shown in Fig. 7d, the adsorption process occurs upon two distinct stages for all tested nanomaterials.^54,55^ The first stage represents the transport of IC molecules from the bulk solution through the boundary layer to the surface of nanomaterials,^56^ whereas the second stage represents the equilibrium condition in which intra-particle diffusion becomes stalled due to a low IC concentration.^56^

### 3.4. Adsorption equilibrium study

**Fig. 8:**
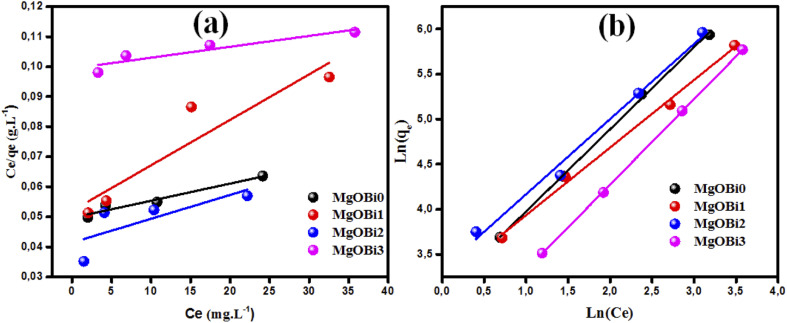
Assessment of isotherm models for IC dye adsorption onto Bi_2_O_3_ doped MgO nanomaterials: (a) Langmuir, (b) Freundlich.

Adsorption isotherms are typically used to characterize the interaction between the adsorbent and adsorbate at equilibrium. The Freundlich and Langmuir isotherms are compared to the experimental data for IC dye adsorption on the obtained nanomaterials. The Langmuir isotherm model implies that the adsorbate covers the adsorbent surface in a monolayer and that adsorption happens uniformly.^59,60^ The Freundlich isotherm model relies on adsorption over a heterogeneous surface with varying adsorption energy and is referred to a multilayer adsorption.^61^ The formulas of the applied isotherms models and the computed parameters are displayed in Table 4. The findings of IC dye adsorption are shown in Fig. 8 and Table 4.

**Table 4:** Isotherm models for IC dye sorption Bi_2_O_3_ doped MgO nanomaterials

Equilibrium model	Linear form	Nanomaterials	Parameters	Values
Langmuir^62^	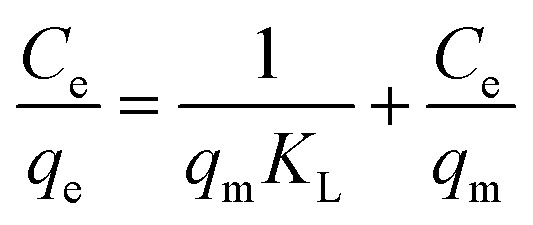	MgOBi0	*q*_m_ (mg g^−1^)	126.00
			*K*_L_ (mg g^−1^)	0.19
			*R*_L_ (L mg^−1^)	0.05
			*R* ^2^	0.96
		MgOBi1	*q*_m_ (mg g^−1^)	66.22
			*K*_L_ (mg g^−1^)	0.17
			*R*_L_ (L mg^−1^)	0.06
			*R* ^2^	0.94
		MgOBi2	*q*_m_ (mg g^−1^)	276.00
			*K*_L_ (mg g^−1^)	0.12
			*R*_L_ (L mg^−1^)	0.07
			*R* ^2^	0.96
		MgOBi3	*q*_m_ (mg g^−1^)	66.22
			*K*_L_ (mg g^−1^)	0.15
			*R*_L_ (L mg^−1^)	0.06
			*R* ^2^	0.95
Freundlich^63^	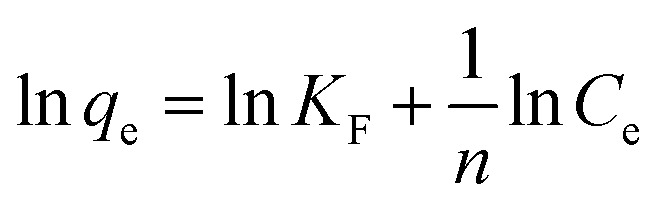	MgOBi0	*n*	1.10
			*K*_F_ (L mg^−1^)	21.46
			*R* ^2^	0.99
		MgOBi1	*n*	1.33
			*K*_F_ (L mg^−1^)	24.17
			*R* ^2^	0.99
		MgOBi2	*n*	1.19
			*K*_F_ (L mg^−1^)	27.93
			*R* ^2^	0.99
		MgOBi3	*n*	1.05
			*K*_F_ (L mg^−1^)	10.76
			*R* ^2^	0.99

The linear regression coefficient *R*^2^ obtained from the Freundlich isotherm model for all used nanomaterials is greater than that obtained from the Langmuir model, indicating that the Freundlich isotherm model fits the experimental data very well. The *n* value determines the type of isotherm; for example, if 1/*n* equals zero, 0 < 1/*n* < 1, or 1/*n* > 1, the isotherm is unfavorable, favorable, or irreversible, respectively.^61^ The present investigated data *n* values are 1.10, 1.33, 1.19, and 1.05 for MgOBi0, MgOBi1, MgOBi2, and MgOBi3, respectively, indicating that the adsorption is favorable.

The maximum adsorption capacities of several adsorbents reported for the IC elimination are presented in Table 5. As shown, the currently described MgBiO2 nanosorbent possesses the remarkable adsorption capacity to remove IC dye from wastewaters, demonstrating its potential utility.

**Table 5:** Comparison of IC adsorption capacities for several sorbents

Adsorbents	Synthesis methods	Surface area (m^2^ g^−1^)	Adsorption capacity (mg g^−1^)	Ref.
MgFe_2_O_4_	Sol–gel	28.8	46	64
Activated carbon	Commercial	1250.3	79.49	65
Mesoporous Mg/Fe	Sol–gel	85.6	62.8	66
Carbon nanotube	Chemical vapor deposition	74.2	88.5	67
COOH–CNT	Chemical vapor deposition	145.9	136	67
Silver nanoparticle	Green reduction	—	73.05	68
Fe_3_O_4_ nanoparticles	Bio-synthesis	78.61	99.71	69
Cobalt hydroxide nanoparticles	Co-precipitation	—	62.5	70
**MgOBi2**	**Co-precipitation**	**12.2**	**126**	**Current work**

### 3.5. Thermodynamics studies

**Fig. 9:**
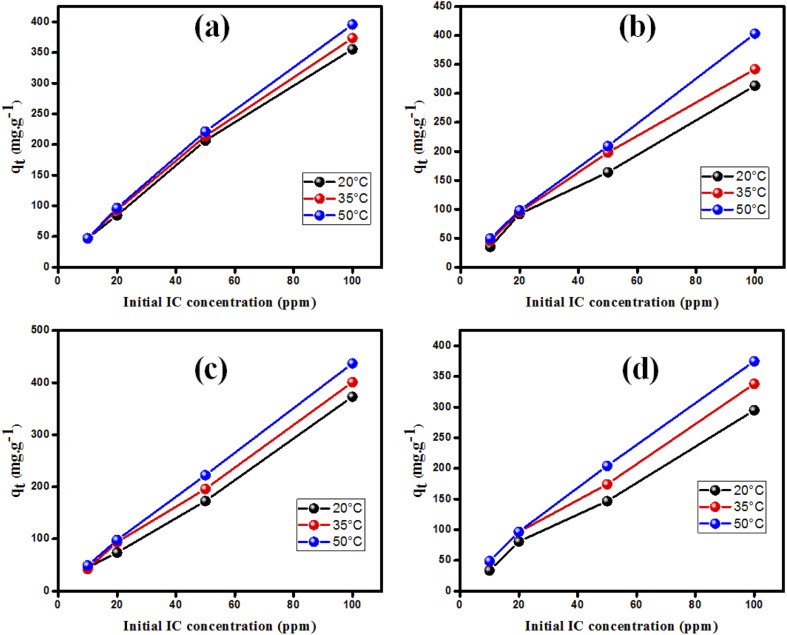
The temperature impact on the adsorption of IC using different IC concentrations on (a) MgOBi0, (b) MgOBi1, (c) MgOBi2, and (d) MgOBi3.

Fig. 9 illustrates that raising the solution temperature from 20 °C to 50 °C substantially increased the adsorbed amount of IC on MgOBi0, MgOBi1, MgOBi2, and MgOBi3. A possible explanation could be creating additional adsorption sites through the disintegration of nanoparticles at high temperatures and/or the acceleration of diffusion-controlled mechanisms that control adsorption.^71^

The equilibrium constant *K*_c_ was obtained from the pollutant concentration on sorbent and solution at equilibrium (*C*_ad_ and *C*_e_ in mg L^−1^). The enthalpy (kJ mol^−1^, Δ*H*°) and entropy (kJ mol^−1^, Δ*S*°) values were extracted from plotting (ln *K*_c_) *versus* temperature reciprocal (*T*^−1^) according to eqn (4), and the obtaining were applied in eqn (5) to compute the free energy (kJ mol^−1^, Δ*G*°) (Table 6).4
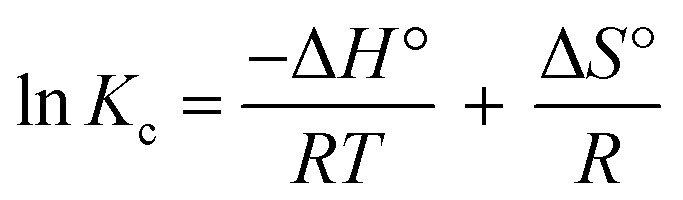

5 Δ*G*° = Δ*H*° − *T*Δ*S*°

**Table 6:** Thermodynamic parameters for the adsorption of IC on MgOBi0, MgOBi1, MgOBi2, and MgOBi3

Fed conc. (mg L^−1^)	Δ*H*° (kJ mol^−1^)	Δ*S*° (kJ mol^−1^)	Δ*G*° (kJ mol^−1^) 293 K	Δ*G*° (kJ mol^−1^) 308 K	Δ*G*° (kJ mol^−1^) 323 K
**MgOBi0**					
10	9.827	0.055	−6.537	−7.086	−7.635
20	60.178	0.248	−13.769	−16.251	−18.732
50	19.124	0.077	−3.832	−4.602	−5.372
100	17.065	0.065	−2.198	−2.844	−3.491

**MgOBi1**					
10	161.792	0.548	−1.586	−7.068	−12.551
20	55.449	0.237	−15.145	−17.514	−19.883
50	39.058	0.137	−1.743	−3.112	−4.481
100	35.703	0.124	−1.112	−2.348	−3.583

**MgOBi2**					
10	81.818	0.288	−4.140	−7.025	−9.909
20	101.992	0.384	−12.518	−16.361	−20.204
50	50.377	0.175	−1.851	−3.603	−5.356
100	33.710	0.122	−2.568	−3.786	−5.003

**MgOBi3**					
10	125.227	0.429	−2.738	−7.033	−11.327
20	71.800	0.287	−13.795	−16.667	−19.540
50	45.175	0.154	−0.777	−2.319	−3.861
100	28.923	0.100	−0.894	−1.894	−2.895

The positive Δ*H*° values indicated that IC adsorption on MgOBi0, MgOBi1, MgOBi2, and MgOBi3 is endothermic. Furthermore, the negative Δ*G*° indicates the spontaneity of the IC adsorption on the four sorbents. The increase of negative Δ*G*° values proportionally with temperature showed that the sorption was favorable.

### 3.6. IC dye adsorption mechanism investigation

**Fig. 10:**
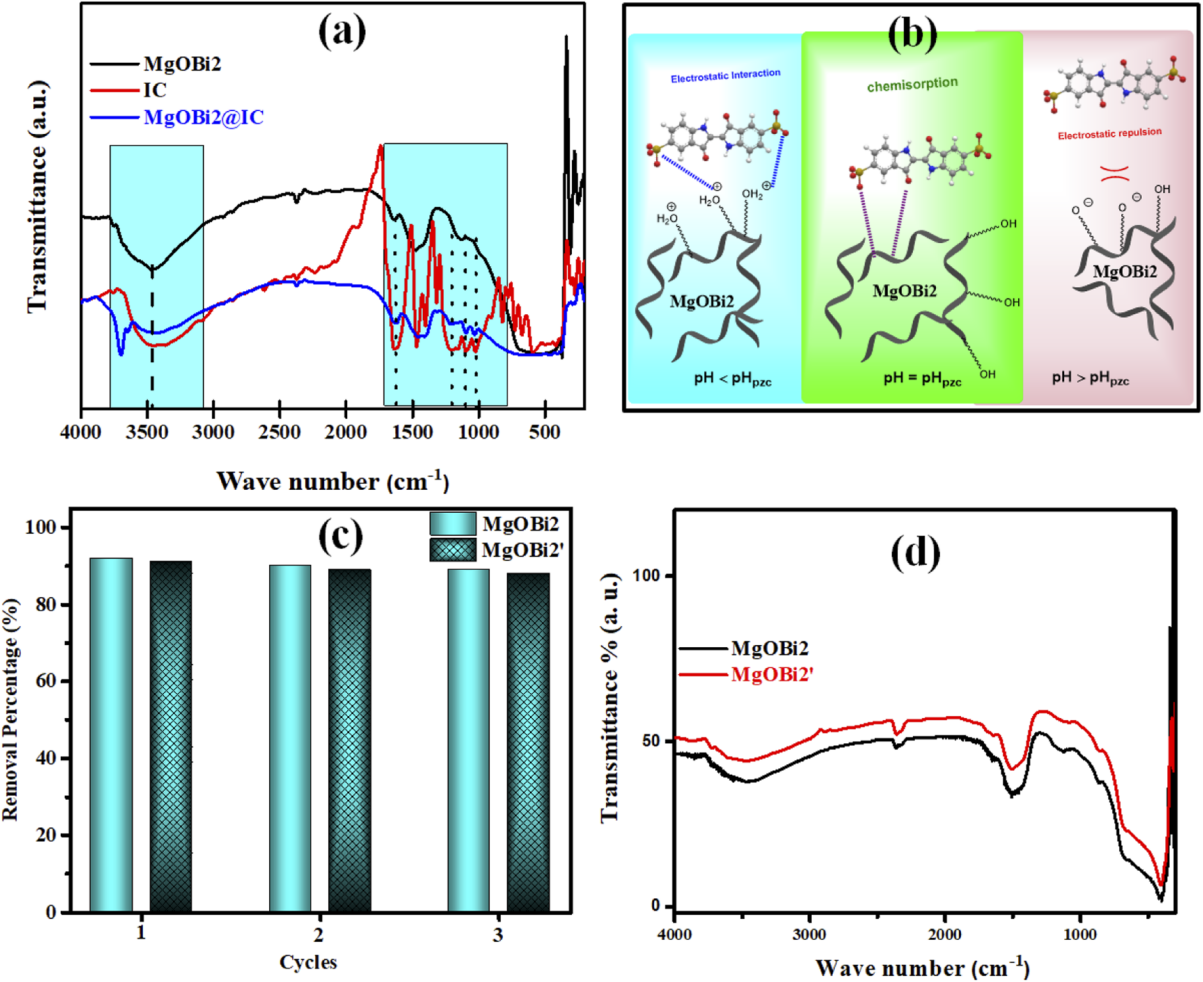
(a) FTIR spectra of IC, MgOBi2 and MgOBi2@IC (b) the proposed adsorption mechanism of IC onto MgOBi2 (c) reusability efficiency of MgOBi2 and used MgOBi2 after three months (MgOBi2′) (d) FTIR spectra of regenerated MgOBi2 and MgOBi2′.

FTIR analysis was used to clarify the IC dye molecule adsorption mechanism onto MgOBi2 nanoparticles. The FTIR spectra of free IC dye molecules, MgOBi2 and MgOBi2@IC are shown in Fig. 10a, and the important FTIR bands are included in Table 7.

**Table 7:** Important FTIR bands for IC, MgOBi2 and MgOBi2@IC

Important bands	IC (cm^−1^)	MgOBi2@IC (cm^−1^)
N–H stretching	3416	3415
C=O stretching	1636	1636
C=C stretching	1469	1421
C–N bonding	1362	1360
SO_3_^−^ stretching	1161, 1098 and 1032	1153, 1096 and 1024

As shown in Table 7 and Fig. 10a, the IC characteristic bands appear in the spectrum of MgOBi2@IC and slightly shift toward a lower wavenumber than in the spectrum of free IC. For example, the SO_3_^−^ stretching bands slightly shift after IC adsorption, owing to the creation of bonds between the oxygen atoms in IC molecules and MgOBi2 nanoparticles. This study established that IC molecules are successfully adsorbed onto the surface of MgOBi2 *via* chemisorption. Fig. 10b depicts the plausible sorption mechanism of IC molecules onto MgOBi2.

### 3.7. Reusability, regeneration and stability studies

The regeneration of the adsorbent is a critical parameter, and its reusability is a necessary economic factor. Then, it is vital to confirm the cyclic availability of MgOBi2. Following the sorption experience, the utilized MgOBi2 nanoparticle was filtered and then calcinated for one hour at 776 K. After that, the recovered MgOBi2 was repurposed. The reusability performance is depicted in Fig. 10c. It was verified that MgOBi2 has effectually involved at least three continual cycles for the IC dye elimination. Furthermore, the long-term stability of the MgOBi2 nanosorbent was investigated after three months. As shown in Fig. 10c, the minor loss in the adsorption capacity for used MgOBi2 after three months (MgOBi2′) can be due to the loss of some adsorbent binding sites and not the structural degradation of nanosorbent. The stability of the MgOBi2 nanosorbent has been checked by FTIR (Fig. 10d). The results demonstrate that the FTIR spectrum of recuperating MgOBi2′ remain unchanged after three months suggesting the structural stability of MgOBi2.

### 3.8. Computational investigation of MgO doping mechanism

**Fig. 11:**
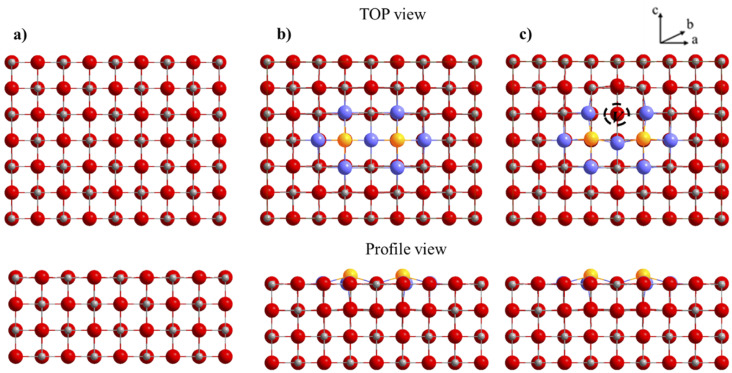
Top and side view for optimized surface slab [200]. (a) The supercell (4 × 3 × 2) of the bare surface, (b) the Bi_2_O_3_ doped surface without the creation of a vacancy, (c) the Bi_2_O_3_ doped surface with vacancy.

To evaluate the effect of dopants on the surface, we retained the surface [200], which is the most stable and one of the most studied for molecules activation. We considered different models of magnesium oxide to study the effect of bismuth(iii) oxide dopants on the structural, energetic and electronic properties of the surface [200]. Compliance with electro neutrality requires the presence of two trivalent dopants for a magnesium vacancy. For this model, a supercell (4 × 3 × 1) is used. Two dopants have been substituted for two Mg on the same upper face of the surface with a magnesium vacancy created on this same face to compensate for the charge difference between Mg^2+^ and Bi^3+^. The thickness of the slab model for this surface supercell is four layers for a total of 192 atoms (96 Mg and 96 O atoms). The bottom three layers were fixed during the optimization (Fig. 11).

The creation of magnesium vacancy leads to significant distortions in the oxygen network in this structure. The dopant atom has moved to coordinate five and forms a short Bi–O bond of length 2.16 Å. The Mg–O bonds are distributed in a range of 1.93 to 2.11 Å. The substitution energy is computed by using the following equation.

6 *E*_sub_ = *E*_Mg_*x*−3_O_*x*_Bi_2__ + 3*E*_Mg_ − 2*E*_Bi_ − *E*_Mg_*x*_O_*x*__

where *E*(Mg_*x*_O_*x*_) is the magnesium oxide surface energy, *E*_Mg_*x*−3_O_*x*_Bi_2__ is the energy of a doped magnesium oxide surface in the presence of a vacancy, *E*_Mg_ and *E*_Bi_ is the energy of a magnesium atom. The values obtained for each dopant are collated in Table 8.

**Table 8:** Values of substitution energies magnesium doped with Bi_2_O_3_ for the different vacancy position

Super cell (4 × 3 × 1)	Vacancy position (Mg^2+^)		
	First neighbor	Second neighbor	Third neighbor
*E*_sub_ (eV)	−2.096	−1.367	−0.884

The results reported in Table 8 indicate a negative substitution energies ranging from −2.096 eV to −0.884 for the first to the third neighbors vacancy respectively. This clearly shows that the substitution is a very favorable thermodynamic mechanism.

**Fig. 12:**
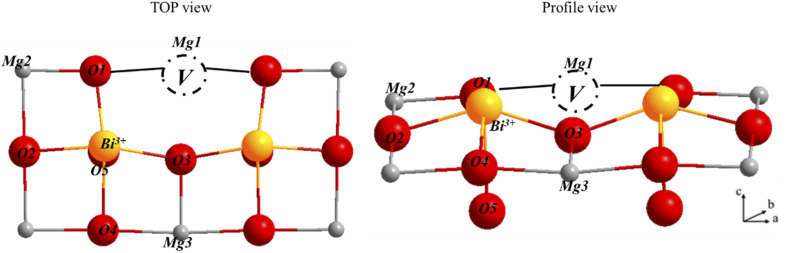
The numbering of atoms that used for Bader charge calculations.

The oxygen nearest neighbors of the dopant cations Mg1, Mg2 and Mg3 have much smaller formation energies than those of the undoped MgO surface, indicating that magnesium vacancy formation is very favorable with dopants. The formation of a vacancy at the second neighbor also requires less energy than on the surface of pure magnesium oxide, although it is more expensive than the first neighbor. Therefore, the vacancy formation energy at the doped surface is lower than that at the pure surface, regardless of the location of the vacancy on the surface. The creation of an oxygen vacancy leads to a redistribution of electron densities in the system. In order to evaluate these redistributions, we calculated the Bader charges for three structures: bare surface, doped surface and doped surface with magnesium vacancy. Fig. 12 shows the atomic charges in the cell (4 × 3 × 1) with a substituted dopant.

The Bader charge calculations for the bismuth atoms in these configurations were carried out first without creating a vacancy and then with a vacancy. The obtained values are shown in Table 9.

**Table 9:** Values of Bader charges on the surface [200] of magnesium oxide bismuth(iii) oxide

	Bader charge							
	O1	O2	O3	O4	O5	Mg1	Mg2	Mg3
Without vac.	−1.78	−1.64	−1.64	−1.58	−1.77	1.88	1.88	1.90
With vac.	−1.47	−1.44	−19.43	−1.46	−1.71	—	0.80	2.04
Difference	0.31	0.20	0.21	0.12	0.06	—	0.08	−0.14

**Fig. 13:**
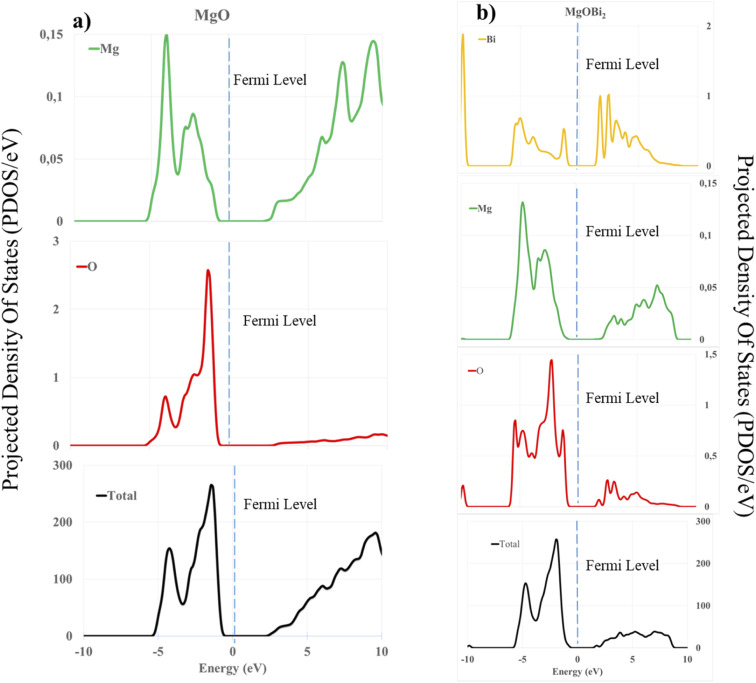
Calculated projected density of states (PDOS) of: (a) O 2sp, Mg 3sp, and total DOS for MgO; (b) O 2sp, Mg 3sp, and Bi 3d, 3f orbitals and total DOS for the doped surface (200).

As expected, we observe that in the absence of Mg^2+^ vacancy, the oxygen and magnesium atoms near the dopant have an increased charge because the dopants can provide fewer electrons than Mg. After creating the vacancy, the charges on the atoms closest to the dopant return to normal values. The positive charges on the Mg and the dopant decrease with the creation of a vacancy because these ions are less solicited to supply electrons to the anions. To better understand the electronic interactions between the surface [200] of magnesium oxide and the dopant bismuth oxide, the PDOS of the surface atoms were calculated (see Fig. 13).

It is observed that the PDOS peaks are mainly composed of 2s, 2p and 3s orbitals for the oxygen atom and 3d and 3f orbitals for bismuth. In the case of the bare surface of MgO, we obtained an energy gap of 3.02 eV, which is comparable to the values obtained in the literature.^72,73^ The s and p orbitals of Mg and O shift to the left, indicating a drop in the energy level in the unoccupied band. Moreover, the peak intensities of the s and p orbitals of Mg appeared after the Fermi level in the valence band (Fig. 13a). This phenomenon justifies the fact that the orbitals concerned participate in the interaction and the lowering of the total energy leading to the stabilization of the surface. Also, a similar observation of lowering and shifting of the s and p orbital of O, Mg and 3d, 3f of Bi, justifies the dopant's participation in the formation of vacancy in the doped surface. According to Fig. 13b, we observe in the two bands (conductions and valences) peaks at the level of each atom, which leads to a decrease in gap energy to 2.15 eV.

## 4. Conclusion

Bi_2_O_3_ doped MgO nanosorbents with various doping concentrations (0, 2.5, 5, and 10%) were successfully prepared by the co-precipitation method. The doping mechanism of MgO by Bi_2_O_3_ is a substitution of three magnesium ions atoms by two bismuth ions, in agreement with XRD study and confirmed using DFT-D3 calculations. Batch experiments revealed that the removal of IC dye by doping MgO dependents to the initial dye concentration, Bi_2_O_3_ doping concentration, and pH values. Bi_2_O_3_ doped MgO prepared at 5% (MgOBi2) is the best adsorbent with a maximum IC adsorption capacity of 126 mg g^−1^ at solution pH equal to seven and contact time of 74 min. Equilibrium and kinetics modeling of the experimental data indicated that the sorption of IC by doped MgO followed the PSO kinetics and Freundlich adsorption isotherm models. The IC adsorption mechanism occurred through the chemisorption process based on FTIR measurements.

## Conflicts of interest

The author(s) declared no potential conflicts of interest with respect to the research, authorship, and/or publication of this article.

## Supplementary Material
